# Outcomes of targeted axillary radiation therapy with omission of axillary dissection in early breast cancer patients with one or two positive sentinel lymph nodes and extracapsular extension

**DOI:** 10.1186/s12893-025-02974-x

**Published:** 2025-06-02

**Authors:** Ahmed Orabi, Asmaa G. Ellaithy, Maha Guimei, Maher H. Ibraheem, Waleed Mohamed M. Fadlalla, Mohamed Fathy Abdelfattah Abdelrahman Elithy, Yasmine Hany Abdel Moamen Elzohery, Ahmed S. Abdelmomen, Sherif Nasser Taha

**Affiliations:** 1 Surgical Oncology Department, National Cancer Institute, Cairo University, Cairo, Egypt; 2 Radiation Therapy Department, National Cancer Institute, Cairo University, Cairo, Egypt; 3 Associate Professor of Pathology, Faculty of Medicine, Alexandria University, Cairo, Egypt; 4 Lecturer of surgical oncology, Faculty of Medicine, Al Azhar University, Cairo, Egypt; 5 Associate Professor of General Surgery, Faculty of Medicine, Ain shams university, Cairo, Egypt; 6 Baheya Centre for Early Detection and Treatment of Breast Cancer, Cairo, Egypt

**Keywords:** Breast cancer, Sentinel lymph node, Extracapsular extension, Axillary lymph node dissection.

## Abstract

**Purpose:**

Axillary dissection has been shown to be equivalent to axillary radiotherapy in the AMAROS trial; however, extracapsular invasion of sentinel lymph nodes was not considered among the evaluated variables. The clinical significance of extracapsular extension (ECE) in one or two positive sentinel lymph nodes remains under investigation. This study aims to evaluate the impact of targeted axillary radiation therapy while omitting completion axillary lymph node dissection (ALND) in the presence of extracapsular extension.

**Methods:**

A retrospective study was conducted between 2016 and 2023 involving cT1–2N0 breast cancer patients who did not receive neoadjuvant chemotherapy and underwent either breast-conserving surgery or mastectomy, with extracapsular extension present in one or two positive sentinel lymph nodes.

**Results:**

Our study included 213 patients treated between 2016 and 2023, with a median follow-up of 48.07 months (range: 9.07–103.10 months). ECE was ≤ 2 mm in 201 patients (94.4%) and > 2 mm in 12 patients (5.6%). A total of 112 patients (52.6%) underwent completion ALND. Systemic recurrence occurred in 24 patients (11.3%), while local recurrence occurred in one patient (0.5%). The 5-year disease-free survival (DFS) rates were 86% in the completion axillary clearance (AC) group and 89% in the non-AC group. The estimated DFS rates for the entire study at 1, 3, and 5 years were 97%, 89%, and 86%, respectively.

**Conclusions:**

Within this single-institution study of early breast cancer patients with predominantly luminal A subtype and mostly limited ECE (≤ 2 mm) treated with targeted axillary radiation, omission of ALND did not result in inferior DFS compared to completion ALND. However, these findings are preliminary, hypothesis-generating, and limited by the retrospective design, short follow-up, and specific patient population studied. Prospective studies are needed to confirm these observations.

**Trial registration:**

Retrospectively registered after the approval of Baheya Ethical Committee, IRB no. 202,304,030,017.

## Introduction

Axillary lymph node status is an important prognostic factor in breast cancer [1]. It is a strong predictor of both recurrence and survival [2]. In patients with negative axilla, sentinel lymph node biopsy (SLNB) has replaced axillary lymph node dissection (ALND) without compromising oncological safety or survival [1, 2]. The AMAROS trial, which included both breast-conserving surgery and mastectomy, demonstrated that axillary radiation therapy is equivalent to completion ALND in these patients [3].

According to the National Comprehensive Cancer Network (NCCN) Guidelines, no further axillary surgery is recommended in early breast cancer only when the following criteria are met: T1–T2 tumors, pN1mi disease, no neoadjuvant chemotherapy, and planned adjuvant radiotherapy [4]. Consequently, extracapsular extension (ECE) is considered a high-risk feature that may favor completion ALND.

In early breast cancer, the number of positive axillary lymph nodes plays a key role in determining axillary management. A low nodal burden is defined as one or two positive lymph nodes, whereas three or more positive nodes indicate a high nodal burden [1, 2]. Ultrasound imaging has a critical role in predicting axillary nodal burden; however, early nodal metastases may not induce structural changes detectable by ultrasound [1]. Additionally, clinicopathological characteristics and the biological behavior of the tumor can significantly influence the extent of nodal involvement [5–8].

Extracapsular extension refers to the spread of neoplastic cells beyond the nodal capsule. Its impact on locoregional or systemic recurrence, however, remains unclear [9]. Several studies have investigated the prognostic significance of ECE [10–17]. It has been suggested that ECE is associated with increased axillary nodal burden and may negatively affect disease-free survival (DFS) and overall survival (OS) [10–12]. Some researchers have also reported that the extent (diameter) of ECE may have prognostic implications [15–17].

This study aims to investigate the clinical significance of ECE and evaluate the outcomes of omitting completion ALND in favor of targeted axillary radiation therapy in patients with early-stage breast cancer. This analysis is exploratory in nature and reflects a single institution’s experience, intended to generate hypotheses rather than provide definitive evidence for altering clinical practice.

## Methods

A single-institution retrospective study was conducted between 2016 and 2023, including patients with cT1–2N0 breast cancer who did not receive neoadjuvant chemotherapy and underwent either breast-conserving surgery or mastectomy, with ECE identified in one or two positive sentinel lymph nodes. Exclusion criteria included patients with T3 or T4 tumors, clinically positive axilla, more than two positive sentinel lymph nodes, or those who received neoadjuvant chemotherapy.

Patient allocation to either completion ALND or observation with radiation therapy was not randomized. Initially, ECE was considered a high-risk feature, and completion ALND was the standard of care. However, following the emergence of data supporting the omission of axillary dissection, the management approach shifted towards regional nodal irradiation.

After obtaining approval from the Baheya Ethical Committee (IRB No. 202304030017), data were collected for each patient, including demographic information, histopathological type, tumor grade, and molecular subtype. Molecular subtypes were defined as follows:

- **Luminal A**: Estrogen and/or progesterone receptor-positive, HER2/neu-negative, and Ki-67 < 15%.
- **Luminal B**: Estrogen and/or progesterone receptor-positive, HER2/neu-negative, and Ki-67 ≥ 15%.
- **HER2-enriched**: HER2/neu overexpression with variable hormone receptor status.
- **Triple-negative**: Negative for estrogen, progesterone, and HER2/neu receptors.

Additional data collected included TNM stage, size of ECE, type of breast surgery, adjuvant treatment, DFS, and incidence of locoregional or systemic recurrence. OS was not assessed due to the relatively short follow-up period. Cox regression analysis was performed to determine which variables independently affected DFS in the study population.

### Histopathological evaluation

Histopathological examination of hematoxylin & eosin-stained sections of axillary lymph nodes was performed by a senior breast pathologist. The cases that showed lymph node involvement were further evaluated for two parameters.

#### Focal versus diffuse nodal involvement

The first parameter evaluated is whether the nodal involvement by metastasis was focal versus diffuse. Focal involvement was defined as metastatic deposits occupying ≤ 25% of nodal size and diffuse involvement was assigned when > 25% of the lymph node was occupied by tumor, regardless of the lymph node size.

#### The size of extranodal extension (using 3D HISTECH scanner)

The second parameter was the size of the ECE measured in mm from the outer surface of the nodal capsule.

The 3D HISTECH scanner was used to scan the entire hematoxylin & eosin-stained sections and turn them into high-resolution, digitized images. The digital images are captured in successive layers to create a 3D view of the tissue architecture.

**Fig. 1:**
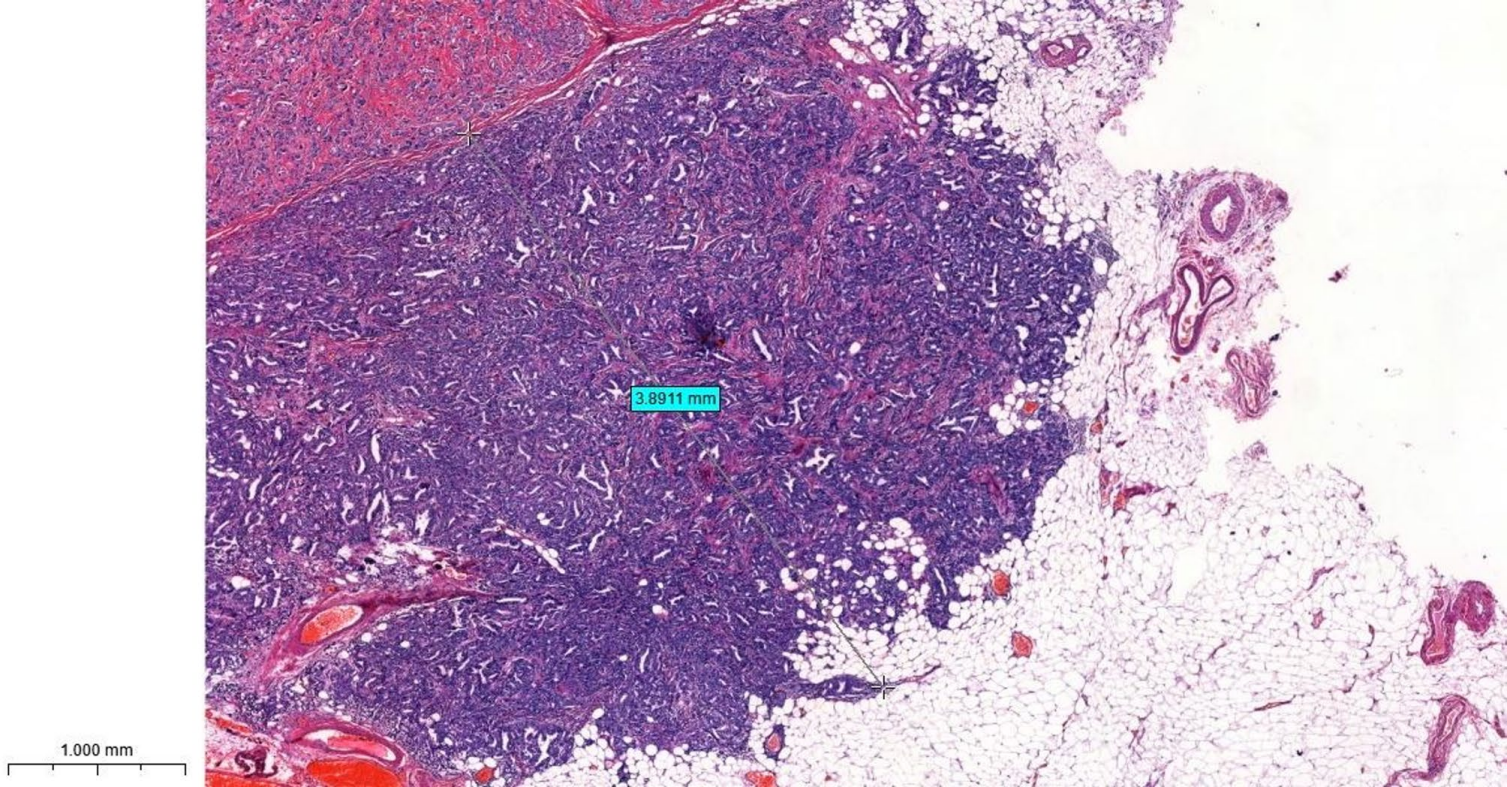
An axillary lymph node showing diffuse involvement of the nodal tissue with extensive extranodal infiltration beyond the nodal capsule. The size of extranodal extension is measured as shown in the figure from the outer surface of the capsule to the furthest away tumor cells. In this case it is equal to 3.89 mm

Using a specialized software, the precise measurement of ECE was obtained by measuring the size of the tumor tissue from the outer surface of the nodal capsule to the furthest cluster of tumor cells away from the lymph node and was classified as limited (≤ 2 mm) and extensive (> 2 mm) (Fig. 1).

### Statistical methods

SPSS Statistics for Windows version 27.0 was used for data management and analysis. Disease-free survival was calculated from the date of surgery to date of recurrence (local/systemic or both). Both life tables (actuarial) and Kaplan-Meier estimated survival and log-rank tests were used to compare survival curves. The p value was set to be significant at the 0.05 level.

## Results

Our study included 213 patients, with a median follow-up of 48.07 months (range: 9.07–103.10 months). The mean age was 55 ± 11.5 years (range: 31–85 years). A total of 79 patients (37.1%) were premenopausal, while 134 patients (62.9%) were postmenopausal. Mastectomy was performed in 153 patients (71.8%), and breast-conserving surgery in 60 patients (28.2%). Invasive ductal carcinoma was the most common histopathological type, observed in 149 patients (70%). Tumor grade 2 was reported in 149 patients (70%). Luminal A was the predominant molecular subtype, accounting for 93.9% of cases. One positive SLN was found in 114 patients (53.5%), while 99 patients (46.5%) had two positive SLNs. ECE was ≤ 2 mm in 201 patients (94.4%) and > 2 mm in 12 patients (5.6%). All patient’ and tumor characteristics are summarized in Table 1.

**Table 1:** Patients’ and tumor characteristics

Number = 213			
Age	Mean	Std. Deviation	Median
	55	11.51	54
Family history	Positive/Negative	Number	Percentage
	Yes	51	23.9%
	No	162	76.1%
Menopause	Premenopausal	79	37.1%
	Postmenopausal	134	62.9%
Tumor stage	T1	81	38%
	T2	132	62%
Molecular subtype	Luminal A	200	93.9%
	Luminal B	10	4.7%
	Triple negative	3	1.4%
Histology	IDC	149	70%
	ILC	15	7%
	Mixed IDC and ILC	35	16.4%
	Others	14	6.6%
Grade	1	31	14.5%
	2	149	70%
	3	33	15.5%
Extensive intraductal component	No	53	24.9%
	Yes	160	75.1%
Number of positive nodes	1	114	53.5%
	2	99	46.5%
Extranodal extension in mm	≤ 2 mm	201	94.4%
	> 2 mm	12	5.6%
Focal or diffuse extension	Focal	88	41.3%
	Diffuse	125	58.7%

**Fig. 2:**
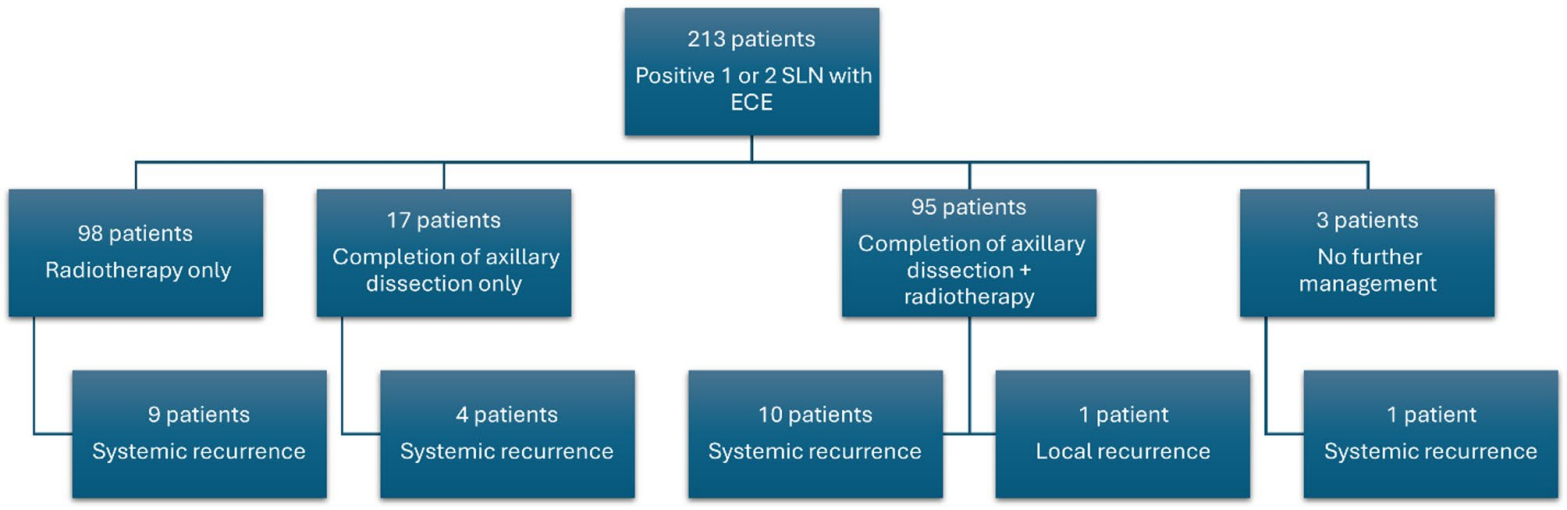
Flowchart illustrates axillary management and pattern of recurrences

Completion ALND was performed in 112 patients (52.6%), of whom 95 patients (44.6%) also received radiation therapy. A total of 98 patients (46%) received adjuvant radiation therapy alone (Fig. 2). All patients who underwent breast-conserving surgery received whole breast radiation therapy, including additional axillary radiation. Among those who underwent mastectomy, 133 patients (62.4%) received chest wall and additional axillary radiation. However, 20 patients (9.4%) did not receive axillary radiation therapy. Whole axillary radiation therapy was given to 66 patients (31%), and level III radiation therapy was administered to 127 patients (59.6%). Adjuvant chemotherapy was administered to 170 patients (79.8%), and hormonal therapy was given to 210 patients (98.6%) (Table 2).

**Table 2:** Treatment characteristics

Surgery type	Wide local excision	60	28.2%
	Mastectomy	153	71.8%
Adjuvant chemotherapy	Yes	170	79.8%
	No	43	20.2%
Adjuvant hormonal therapy	Yes	210	98.6%
	No	3	1.4%
Axillary dissection versus radiation therapy only	Radiotherapy only	98	46%
	Axillary dissection	112	52.6%
	No radiotherapy or axillary dissection	3	1.4%

**Fig. 3:**
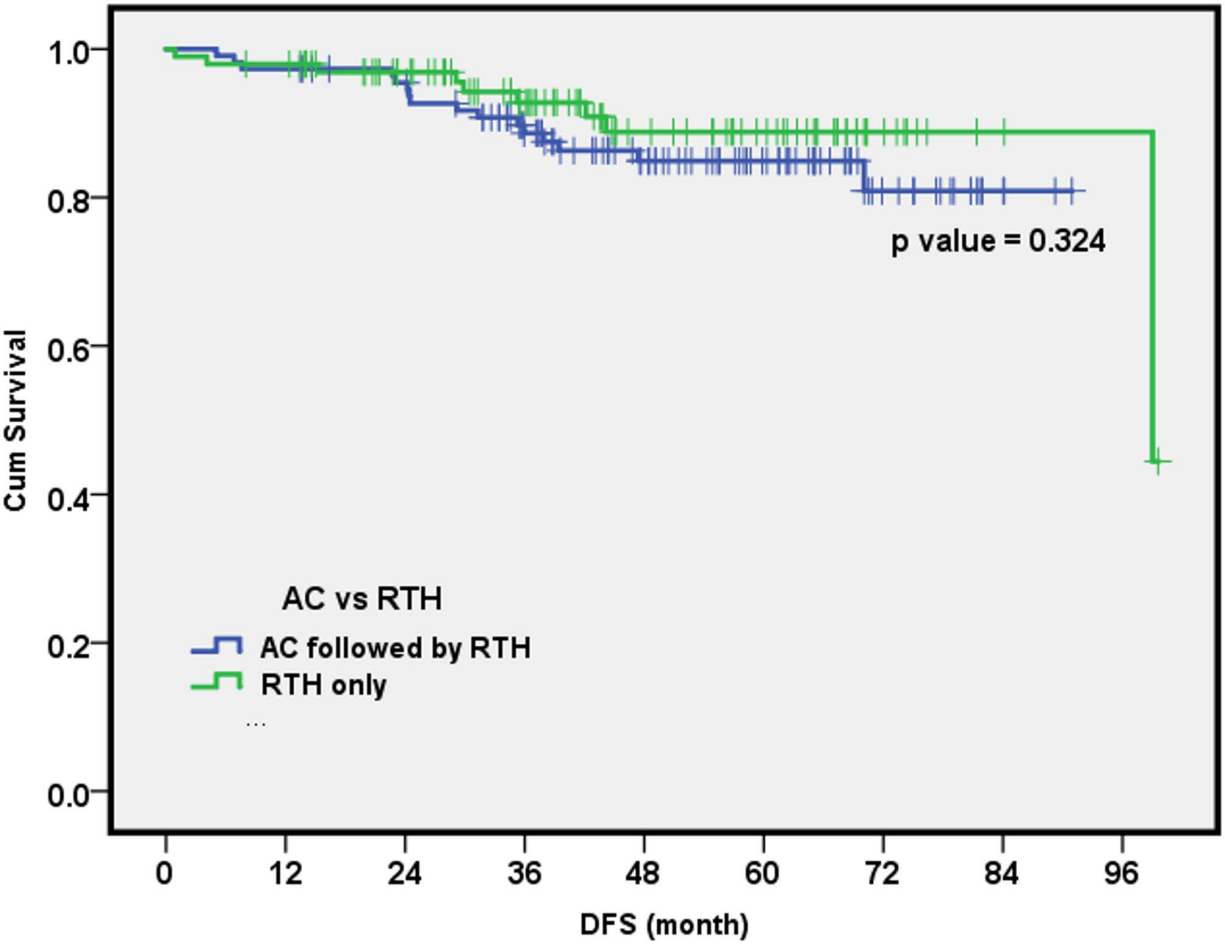
Disease-free survival cumulative rate for axillary clearance (AC) followed by radiotherapy versus radiotherapy alone

The DFS estimates for the entire study at 1, 3, and 5 years were 97%, 89%, and 86%, respectively. Recurrence was observed in 25 patients (11.7%). Of these, 24 patients (11.3%) experienced systemic recurrence, while 1 patient (0.5%) developed local recurrence. The regional recurrence rate was 0%. The 5-year DFS rates for patients who underwent completion axillary clearance (AC) compared with those who received radiation therapy alone were 86% and 89%, respectively (Fig. 3).

**Fig. 4:**
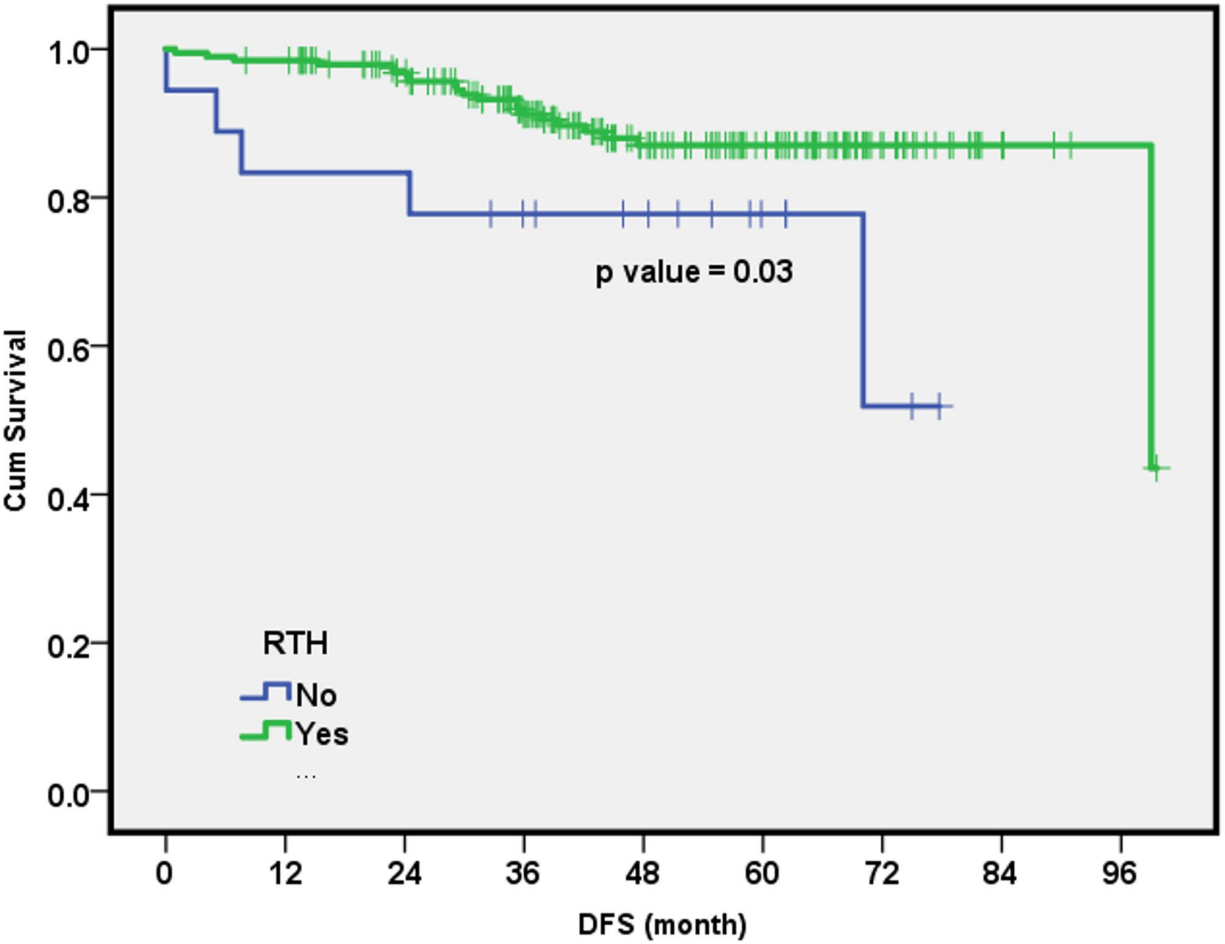
Disease-free survival cumulative rate of patients receiving adjuvant radiation therapy

Cox regression analysis was performed, including the following variables: age (≤ 40 or > 40 years), whether AC was followed by radiation therapy or not, ECE size (≤ 2 mm or > 2 mm), extension type (focal or diffuse), and radiotherapy treatment (yes/no). The results suggested that young age was an independent factor affecting DFS. Specifically, women aged ≤ 40 years were approximately 10 times more likely to experience recurrence compared to older women (HR, 95% CI = 9.84 (1.85–52.3), p value = 0.007). Additionally, women who did not receive radiotherapy may have had a substantially higher risk of recurrence, potentially more than 10 times greater than those who received radiation therapy. (HR, 95% CI = 10.35 (1.87–57.4), p value = 0.03) (Fig. 4).

## Discussion

Patients with ECE were not analyzed in the AMAROS study [9]. Several studies have explored whether the application of these results could be extended to patients with higher risk factors, such as the presence of ECE in positive sentinel lymph nodes [10–17].

The correlation between ECE in sentinel lymph nodes and metastasis in non-sentinel nodes has been examined by many authors. In a study included 402 patients, all of whom with positive sentinel lymph nodes underwent axillary dissection, 158 patients (39.3%) had ECE and high nodal burden [12]. In another study by Schwentner et al., it was concluded that there was an increase in nodal burden following axillary dissection [13]. Vane et al. demonstrated that the presence of ECE was associated with ≥ 4 positive nodes in axillary dissection (odds ratio 2.8, 95% CI 1.936–4.270, *p* < 0.001). However, they also reported that the presence of ECE was not an independent risk factor for either DFS or OS [14]. In our study, 112 patients (52.6%) underwent completion axillary dissection, and 6 patients (2.8%) had positive non-sentinel nodes. Only 1 patient (0.5%) had more than 4 positive nodes.

The prognostic significance of ECE diameter remains a topic of debate in the literature. Some studies have reported that ECE larger than 2 mm is associated with higher rates of locoregional recurrence and poorer survival, while smaller ECE (< 2 mm) is often considered biologically similar to ECE-negative disease [15–17]. However, in a study by de Freitas et al. that included 65 patients with ECE (44.6% luminal A), no significant difference in DFS or OS was found between patients with smaller and larger ECE after ten years of follow-up [10]. Similarly, a study of 103 patients with ECE (25.2% luminal A) reported that those with ECE > 2 mm had a higher axillary nodal burden but did not necessarily experience worse long-term survival [11]. In another study, XiaoXi et al. evaluated 158 patients with ECE (22.1% luminal A) and found no significant prognostic impact when comparing patients with ECE above and below the 2 mm threshold [12]. In our study, which included a majority of luminal A patients (93.9%), we observed that there was no relationship between the diameter of ECE and DFS (*p* = 0.24). However, this analysis was based on only 12 patients with ECE > 2 mm, which limits statistical power and may obscure potential differences.

In a study by Van et al., 741 patients with ECE, including 484 patients (65.3%) with luminal A subtype, underwent completion axillary dissection. Local recurrence occurred in 1.6% of patients with ECE, compared to 2.6% of those without ECE, while regional recurrence rates were 0.9% and 1.1%, respectively. Distant metastasis was more common in the ECE group (12.2% versus 7.8%), and the 5-year DFS rate was 86.4% in patients with ECE compared to 88.8% in those without ECE [14]. Consistent with these findings, our study included 213 patients, 93.9% of whom had luminal A subtype. We observed local recurrence in 1 patient (0.5%) and systemic recurrence in 24 patients (11.3%), with a 5-year DFS rate of 86%. These findings suggest that ECE is a risk factor for distant metastasis rather than locoregional recurrence.

A retrospective study reported that only 21% of patients received breast and nodal radiation; their patients received whole breast radiation without nodal radiation in 17% (prone radiation) to 28% (supine tangent radiation) who had microscopic extracapsular extension in their sentinel lymph nodes, and they showed a low nodal recurrence rate [18]. Another prospective study reported by Barrio et al. highlighted the impact of microscopic ECE on recurrence. Nodal radiation was given in 39% and 17% of patients with and without microscopic ECE, respectively. There was no difference in regional recurrence between both groups without axillary dissection. The 5-year nodal recurrence was only 1.5% [19]. In our study, 193 patients (91%) received nodal radiation. Sixty-six patients (31%) and 127 patients (59.6%) received whole axilla and level III radiation, respectively. The 5-year DFS for patients who received radiation therapy was 87%. P value 0.035. It is important to note that a small subset of patients (approximately 9.4%) did not receive radiation therapy, which may have diluted the observed effects of axillary radiation therapy, potentially resulting in an underestimation of its true impact on regional disease control and survival.

Regarding the levels of axillary radiation, results from Gebhardt et al. reported that 19 patients (18.1%) received whole breast radiation alone, and 86 patients (81.9%) were treated with modified tangent (MT) fields, including levels I/II. Thirty-three patients (31.4%) also received a 3rd supraclavicular node (SCN) directed field. There was no difference in DFS between the MT and SCN fields [20]. Our results suggested that there was no difference in the DFS between level III or whole axillary radiation, p value = 0.416.

In our study, twenty patients who underwent mastectomy (9.4%) did not receive adjuvant radiation therapy. Of these, seventeen patients (8%) underwent completion ALND only. This decision was made during the COVID-19 period when these patients were considered low-risk, and radiation therapy was omitted. Three patients did not receive further axillary treatment: two were unfit for re-surgery or radiotherapy, and the third developed systemic metastasis while receiving adjuvant chemotherapy.

The NCCN guidelines strongly recommend post-mastectomy radiation therapy (PMRT) to the chest wall for patients with 1–3 positive axillary nodes. At our center, we adhere to this recommendation. Our protocol mandates axillary-directed radiotherapy for all patients, utilizing a dedicated axillary and supraclavicular third field, in addition to the standard tangential breast fields.

Since our study specifically focused on patients with initially negative axillary ultrasound who were subsequently found to have limited nodal metastasis with ECE, it is important to consider the known false-negative rate of axillary ultrasound and the contributing tumor-related factors. In the literature, the negative predictive value (NPV) of ultrasound for predicting negative axillary lymph node status varies from 68 to 98% [21, 22], and its accuracy ranges from 66 to 90.2% [5, 6]. These values increase to 68-98.4% for NPV and 81.2-98.5% for accuracy, respectively, in cases of high nodal burden (metastases in ≥ 3 lymph nodes) [5, 6, 21, 22]. In a study reported by Shao et al., 904 patients (71.0%) had negative axilla on ultrasound, and limited nodal burden was found in 761 patients (84.2%) in the postoperative pathology [1]. However, another study involving 2059 patients showed that the sensitivity and specificity of ultrasound for predicting negative axillary lymph node metastasis were 79% (95% CI 75–82%) and 100% (95% CI 99–100%), respectively, with a false-negative rate of 21% (95% CI 17–25%) [2].

Several studies have evaluated the association between various factors and false-negative results, including histological type, tumor size, lymphovascular invasion (LVI), and Her2 status [5–8]. False-negative results were more frequently observed in cases of lobular carcinoma [5]. In our study, most cases were invasive ductal carcinoma (70%), suggesting that other factors may be involved.

Larger tumor sizes are more often associated with increased false-negative results [6, 7]. LVI is more likely to occur in the false-negative group compared to the true-negative group (44% versus 8%, *p* < 0.0001) [7]. In another study, false-negative results occurred only in the LVI-positive group [8]. Her2 status also affects false-negative results; patients with negative Her2 expression are more likely to experience false-negative results compared to those with positive expression (10.5% versus 1.2%, *p* < 0.05) [8]. In our study, we hypothesize that the presence of positive axillary nodes with ECE, despite negative findings on clinical examination and ultrasound at the initial diagnosis, may be attributed to several factors. Most of our patients (62%) had T2 tumors, 89% had positive LVI, and all patients had negative Her2 expression. Additionally, the majority of our patients (94.4%) had nodal microscopic ECE.

Twenty-five patients (11.7%) in our study experienced disease relapse. Among the patients who underwent completion ALND, fourteen (6.6%) developed distant metastasis, and one (0.5%) patient experienced local failure. Notably, more than 50% of the relapses occurred despite axillary clearance, suggesting that completion ALND may not confer a disease-free survival benefit.

While some guidelines recommend ALND in the presence of ECE in a sentinel node [4], our findings suggest that this may not be necessary if axillary radiation is administered. Notably, none of our patients developed isolated axillary nodal recurrence. The targeted axillary radiation approach we adopted may have played a significant role in preventing such recurrences.

This study has several important limitations that should be considered when interpreting the findings. First, as a retrospective, non-randomized analysis, it is inherently vulnerable to selection bias and residual confounding, despite efforts to adjust for known variables. This design precludes definitive causal interpretations, as unmeasured factors may still have influenced the observed outcomes. Second, the single-institution setting limits the generalizability of our findings, as patient characteristics, surgical approaches, and radiotherapy protocols can vary significantly across institutions. Third, the study cohort predominantly comprised patients with luminal A subtype breast cancer, which restricts insights into recurrence patterns and treatment responses in higher-risk subgroups. Fourth, the median follow-up of 48 months may be insufficient to fully capture late recurrences, particularly in hormone receptor-positive breast cancer, where events often occur beyond 5–10 years, potentially underestimating the long-term effects of omitting ALND. Fifth, only 12 patients had ECE > 2 mm, limiting the statistical power to assess its prognostic significance. Sixth, approximately 9.4% of patients did not receive axillary radiotherapy despite the omission of ALND, introducing additional variability that could influence our findings. This inconsistency may lead to an underestimation or overestimation of the impact of axillary radiation therapy on regional control and overall outcomes. Finally, OS was not assessed due to insufficient follow-up and competing risk events, limiting conclusions to recurrence-related endpoints.

Despite these limitations, our findings may provide preliminary data for the feasibility and safety of omitting axillary dissection in this patient group, potentially reducing surgical morbidity without compromising local control. Given the evolving role of radiotherapy in managing the axilla, these results may contribute to the growing body of literature aiming to personalize and de-escalate surgical treatment for early breast cancer.

These limitations necessitate the need for caution in interpreting these findings and highlight the importance of validating these results in larger, prospective, and randomized studies.

## Conclusion

The presence of ECE is associated with a higher risk of distant metastasis rather than locoregional failure. In this retrospective, single-center study composed primarily of patients with luminal A subtype and limited ECE (≤ 2 mm), the addition of targeted axillary radiation therapy appeared to achieve regional control without the need for completion ALND. However, these findings should be interpreted with caution due to several limitations, including the non-randomized design, limited generalizability to other molecular subtypes, relatively short follow-up, variability in radiation protocols, and small number of patients with extensive ECE. Therefore, this study should be considered exploratory and hypothesis-generating. Larger, prospective randomized trials with longer follow-up are essential to validate these observations before practice change can be recommended.

## Data Availability

All datasets used and analyzed are available from the corresponding author upon reasonable request.

## Abbreviations

ALND: Axillary Lymph Node Dissection
ECE: Extracapsular Extension
SLN: Sentinel Lymph Node
DFS: Disease Free Survival
AC: Axillary Clearance
RTH: Radiation Therapy
OS: Overall Survival
SCN: Supraclavicular Node
MT: Modified Tangent
PMRT: Post Mastectomy Radiation Therapy
